# The epidemiology of cervical cancer among indigenous women living in Latin America: A systematic review

**DOI:** 10.1016/j.pmedr.2024.102955

**Published:** 2024-12-24

**Authors:** Iria Riberio Novais, Camila Olegario Coelho, Carla Fabrine Carvalho, Fernanda Surita, Diama Bhadra Vale

**Affiliations:** a Department of Obstetrics and Gynecology, University of Campinas. Rua Vital Brasil, 80. CEP 13083-888, Campinas, São Paulo, Brazil.

**Keywords:** Cervical Cancer, Indigenous people, Screening, Vulnerable population, HPV infection

## Abstract

**Objective:**

To review the epidemiological evidence of cervical cancer among Indigenous women living in Latin America.

**Methods:**

We conducted a systematic review of the evidence contained in 10 databases spanning 2003–2019. Two reviewers independently compared papers' titles and abstracts against the inclusionary criteria, and a third reviewer resolved discrepancies. Blinded reviewers performed the selection. The articles were organized into the following categories: rates, access, and screening; prevalence of precursor lesions; prevalence and genotypes of Human papillomavirus (HPV); and HPV coinfections.

**Results:**

Of the 874 manuscripts we reviewed, 25 were included in the final analysis. We found that cervical cancer is the leading cancer in terms of incidence and mortality among Indigenous women; it presents in advanced stages and is associated with poor survival rates. The prevalence of precursor lesions was higher in women who were geographically isolated. Screening appears to improve outcomes, but women may experience delays in their diagnosis and treatment. Some studies reported populations with a very high prevalence of high-risk Human papillomavirus (hrHPV), and the most frequent genotypes were not different from those of the general population. *Chlamydia trachomatis* was significantly associated with HPV infection.

**Conclusions:**

The data suggested a lack of indicators regarding cervical cancer and its precursor lesions, HPV infection, and cancer indicators. Health policies should target this vulnerable population.

## Highlights

• Cervical cancer is the leading cancer in incidence and mortality in Indigenous women.
• Diagnosis is in advanced stages, and women may experience poor survival.
• Prevalence of precursor lesions is higher in women who are geographically isolated.
• Screening seems to improve outcomes.
• The most frequent Papillomavirus genotypes are not different from the general population.
• *Chlamydia trachomatis* is significantly associated with Papillomavirus infection.

## 1 Background

Approximately 45 million Indigenous people in Latin America are distributed across 826 communities (Comisión Económica para América Latina y el Caribe CEPAL/Fondo para el Desarrollo de los Pueblos Indígenas de América Latina y el Caribe FILAC, 2020). Those Indigenous people are vulnerable given the historical processes they have experienced since the colonization of the Americas (Comisión Económica para América Latina y el Caribe CEPAL/Fondo para el Desarrollo de los Pueblos Indígenas de América Latina y el Caribe FILAC, 2020). Cancers related to infections are responsible for many deaths of Indigenous people; those diseases include cancer of the stomach, liver, and cervix (Alves et al., 2021).

Cervical cancer is the most prevalent cancer and the leading cancer-related cause of death among Indigenous women (Moore et al., 2014; Borges et al., 2019; Muslin, 2024). This cancer has a long natural history, which makes it possible to screen to identify precursor lesions to ensure timely treatment and inhibit the development of the invasive form. Low- and middle-income regions, such as Latin America, have fragile health systems that make it challenging to adopt effective screening programs, especially among women in vulnerable conditions (Vale et al., 2021; Vale and Teixeira, 2023).

Some people have argued that Indigenous women have a greater immunological vulnerability (Fonseca et al., 2015). Early sexual initiation, early conception, and high fertility rates may contribute to more significant and earlier exposure to Human papillomavirus (HPV) in this population (Schiffman et al., 2016). Sexual contact with non-Indigenous people, in the context of illegal occupation or social vulnerability, may infect previously healthy Indigenous women with sexually transmitted diseases. And limitations in healthcare can accordingly lead to high rates of morbidity and death from cervical cancer (Vale et al., 2021).

However, evidence of cervical cancer in Indigenous populations remains scarce. We conducted a systematic review of quantitative evidence related to the epidemiology of cervical cancer among Indigenous women living in Latin America. The data we obtained can shed light on best strategies to improve access to screening and treatment in this population.

## 2 Materials and methods

We conducted a systematic review aiming to answer the question, “What is the quantitative evidence regarding cervical cancer epidemiology among Latin American Indigenous women?.”

A library professional selected the keywords we used in our search: “Cervical Cancer,” “Cervical Intraepithelial Neoplasia,” and “HPV Infection.” We excluded opinion studies, qualitative studies, and case reports. The main outcomes were the prevalence, incidence, and mortality of cervical cancer and its precursor lesions. Additional outcomes included surrogates of the cervical cancer development, such as HPV, screening, diagnosis, treatment, and survival.

**Fig. 1:**
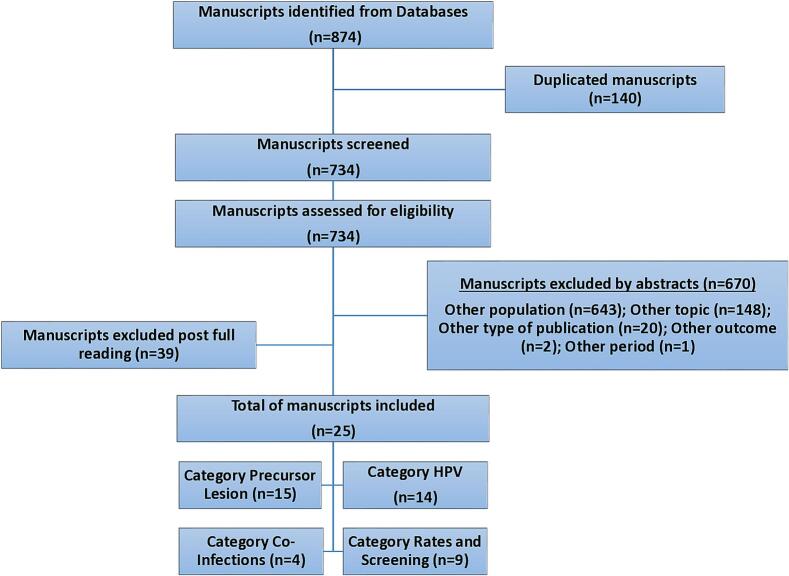
Flowchart of the systematic review of the epidemiology of cervical cancer among Indigenous women living in Latin America.

Two reviewers independently compared the titles and abstracts of the papers on the Rayyan® platform with the inclusionary criteria to select manuscripts. Discrepancies were resolved by a third reviewer. The data collected from the selected manuscripts were systematized in an identification table with key information, such as study location, sample, main results, and conclusions. The articles were organized into topical categories. The first category was “Rates, Access, and Screening,” which reflects the population's risks, access to health care, and screening indicators. The category “Prevalence of Precursor Lesions” pertained to screening test (cytology and histology) results. “HPV Prevalence and Genotypes” included data pertaining to HPV prevalence (overall and high-risk HPV [hrHPV]), genotypes, and variants. The last category, “HPV Coinfections,” included data on sexually transmitted diseases known to facilitate infection or persistence of HPV. Fig. 1 presents the PRISMA flowchart for this systematic review.

This study satisfied the recommendation of the University of Campinas (Campinas, Brazil) to protect the safety and privacy of human subjects. The University of Campinas Ethics Committee approved the study, registered as CAAE: 51446421.9.0000.5404.

### 2.1 Search strategy and selection criteria

We searched the following databases: DecS, Mesh, BVS, Pubmed, Cinahl, Scopus, Web of Science, Embase, Cochrane Library, and Proquest. The search terms included “Cervical Cancer,” “Cervical Intraepithelial Neoplasia,” and “HPV Infection.” We focused on manuscripts published from 2003 to 2019. Studies in English, Spanish, or Portuguese providing quantitative data were eligible for selection. The final reference list was generated based on originality and relevance to the broad scope of this review. We did not use any software to facilitate the synthesis of the evidence reported.

## 3 Results

Of the 874 manuscripts identified in the databases, 140 were duplicates, and 670 were excluded by the abstract review, mainly because they referred to populations other than Indigenous Latin American women. Another 39 manuscripts were excluded after being read fully. The total number of manuscripts included was, therefore, 25 (Table 1).

**Table 1:** The primary characteristics of the studies included in the systematic review of the epidemiology of cervical cancer among Indigenous women living in Latin America.

N	Study	Study Year(s)	Country and Setting	Objective	Subjects (Ethnicity)	Sample	Category
1	Picconi (2003) (Picconi et al., 2003)	Before 2002	Argentina, Puna and Quebrada region (Jujuy Province)	To determine the prevalence of HPV-16 variants (L1 and E6)	Quechua Indians	106 HPV-16 positive samples from 271 women	HPV prevalence and genotypes
2	Tonon (2004) (Tonon et al., 2004)	2002 to 2003	Argentina, Region of Missiones, nine Indian settlements	To evaluate the prevalence of HPV and cytological results	Guarani	207 women	Prevalence of precursor lesions; HPV prevalence and genotypes
3	Brito (2006) (Brito et al., 2006)	2000	Brazil, Pará (Brazilian Amazon), Parakanã community	To evaluate the HPV and STD profile in Parakanã women	Parakanã	49 of all 142 Parakanã women over ten years old	Prevalence of precursor lesions; HPV prevalence and genotypes
4	Tonon (2007) (Tonon et al., 2007)	2002 to 2003	Argentina, Region of Missiones, nine Indian settlements	To identify HPV-16 E6 and L1 molecular variants	Guarani	70 HPV-16 positive samples	HPV prevalence and genotypes
5	Speck (2009) (de Speck et al., 2015)	2005 and 2007	Brazil, South Amazon region, Xingu Indigenous Park	Evaluation of Pap testing	Indigenous women living in Xingu Indigenous Park	518 women in 2005; 564 women in 2007	Prevalence of precursor lesions
6	Deluca (2011) (Deluca et al., 2011)	2007 to 2009	Argentina, Formosa	To determine the prevalence and genotypes of HPV	Pilagá aboriginal	227 women	Prevalence of precursor lesions; HPV prevalence and genotypes
7	Pereira (2011) (Pereira et al., 2011)	2004 to 2006	Brazil, Indigenous Reserve of Dourados, villages of Jaguapirú and Bororó	To learn about the prevalence of precancerous and cancerous lesions and coverage of screening	Indigenous women from Jaguapirú and Bororó, Reserve of Dourados	999 cytology from Jaguapirú and 278 cytology from Bororó	Prevalence of precursor lesions; Rates, Access, and Screening
8	Price (2011) (Price and Asgary, 2011)	2008	Honduras, Yamaranguila (western highlands)	Assessing women's health indicators by questionnaires to women	The municipality of Yamaranguila has a “majority Indigenous population”	134 women over 18	Rates, Access, and Screening
9	Blas (2012) (Blas et al., 2012)	2010 and 2011	Peru, Peruvian Amazon	To evaluate the association between HTLV and HPV infections	Shipibo-Konibo	102 HTLV infected women and 205 community-matched HTLV negative controls	Prevalence of precursor lesions; HPV prevalence and genotypes; HPV coinfections
10	Deluca (2012) (Deluca et al., 2012)	2007 to 2009	Argentina, Formosa	To determine the impact of HPV and ChT infections in women	Pilagá aboriginal	227 women	HPV prevalence and genotypes; HPV coinfections
11	Martorell (2012) (Martorell et al., 2012)	2009	Peru, Loretto Department (Bora native settlement) and Iquitos (urban)	To study the prevalence of HPV and cervix lesion	Bora native Peruvian women	202 non-screened screening urban women, 183 urban women referred by previous lesions, 37 Iquitos urban women, 50 Bora native women	Prevalence of precursor lesions; HPV prevalence and genotypes
12	Mendoza (2013) (Mendoza et al., 2013)	2010 to 2011	Paraguay, Department of Presidente Hayes, Indigenous community	To determine the frequency of HPV infection and other genital infections	Maká (Qemkuket community), Nivacle (Novoctas), Sanapaná (Laguna Pato), Enxet South (Maxhawaya and Espinillo), Toba-Qom (Rio Verde and Toba-Qom)	181 women	HPV prevalence and genotypes; HPV coinfections
13	Fonseca (2014) (da Fonseca et al., 2014)	2004 to 2012	Brazil, Northern Amazonian region (Indigenous reference center in Boa Vista, Roraima)	To evaluate the prevalence of precursor lesions	Yanomami and Yekuana; Macuxi and Wapishana. Indigenous women attended the screening in the center.	2757 tests	Prevalence of precursor lesions
14	Pereira (2014) (Pereira et al., n.d.)	2010 to 2013	Brazil, São Paulo city, referral Indigenous Clinic of São Paulo Hospital	To assess sexual health, reproductive health, and socio-cultural aspects of Indigenous women	Indigenous women from diverse ethnicities attending the clinic (35 ethnicities)	90 women	Prevalence of precursor lesions
15	Rodrigues (2014) (Rodrigues et al., 2014)	2006 to 2007	Brazil, Pará e Mato Grosso, Nãncepotiti	To analyze the prevalence of cytological atypia and HPV infection	Panará	86 women	Prevalence of precursor lesions; HPV prevalence and genotypes
16	Fonseca (2015) (Fonseca et al., 2015)	2013	Brazil, Northern Amazonian region (Yanomami Indigenous District; Eastern Indigenous District)	To evaluate the prevalence of precursor lesions and HPV	Indigenous women living in the Yanomami District (Yanomami and Yekuana); or Eastern District (Macuxi and Wapishana)	664 native women (305 Yanomami and 359 Eastern District)	Prevalence of precursor lesions; HPV prevalence and genotypes; Rates, Access, and Screening
17	Mongelos (2015) (Mongelos et al., 2015)	2010 to 2011	Paraguay, Department of Presidente Hayes, Indigenous community	To determine the frequency of HPV genotypes and their association with BVg	Maká (Qemkuket community), Nivacle (Novoctas), Sanapaná (Laguna Pato), Enxet South (Maxhawaya and Espinillo), Toba-Qom (Rio Verde and Toba-Qom)	181 women	HPV prevalence and genotypes; HPV coinfections
18	Speck (2015) (de Speck et al., 2015)	2005 to 2011	Brazil, Mato Grosso, Xingu Indigenous Park	To analyze the abnormal results in young and elderly Indigenous women	Indigenous women from Xingu	1386 tests from women 12–24, 115 from women older than 64	Prevalence of precursor lesions
19	Aguiar (2016) (Aguiar et al., 2016)	2005 to 2015	Brazil, Cancer Clinic, São Paulo	To assess cancer characteristics in treatment patients	Indigenous women referred to special cancer care in a metropolitan center from the ethnicities Tupi-guarani, Pankararu, and Kaiabi	50 Indigenous with solid tumors, 29 of them being women	Rates, Access, and Screening
20	Fuenmayor (2018) (Fuenmayor et al., 2018)	2016	Venezuela, Maniapure region, outpatient clinic	To detect precursor lesions and HPV infection	Women attending the clinic: Creole women (non-indigenous) and Eñepa women (Indigenous)	25 Creole and 35 Eñepa women	Prevalence of precursor lesions; HPV prevalence and genotypes
21	Renna Jr. (2018) (Renna Junior and Silva, 2018)	2000 to 2012	Brazil, Hospital-based cancer registries	To analyze temporal trends and factors related to cervical cancer late stage diagnosis	Diverse	65,843 cervical cancer cases, 143 in Indigenous women	Rates, Access, and Screening
22	Vargas-Robles (2018) (Vargas-Robles et al., 2018)	?	Venezuela, Northern part of the Amazon state	To determine the diversity of cervical HPV types	Piaroa Amerindians living in a gradient of urbanization from rainforest to town and town mestizos	82 Amerindians and 29 mestizos	Prevalence of precursor lesions; HPV prevalence and genotypes; Rates, Access, and Screening
23	Velazquez (2018) (Velázquez et al., 2018)	2015 to 2017	Paraguay, Department of Caaguazu, Primary Health Care	To determine the prevalence of precursor lesions and sexual and reproductive antecedents	Natives of Caaguazu	129 Indigenous women	Prevalence of precursor lesions; Rates, Access, and Screening
24	Borges (2019) (Borges et al., 2019)	2000 to 2012	Brazil, Acre Satet (Amazonian region)	To estimate cancer mortality in the Indigenous population	Indigenous women from Acre State	81 cancer deaths, 12 cervical cancer (C53)	Rates, Access, and Screening
25	Collins (2019) (Collins et al., 2019)	2015	Peru, Lower Napo River region of the Peruvian Amazon	Descriptive analysis of a Demographic and health survey regarding access to healthcare	Indigenous or mixed Indigenous women (*mestizo*)*	121 women	Rates, Access, and Screening


HPV - Human papillomavirus; L1 - Major capsid protein of HPV; E6 - Oncoproteins of high-risk HPV; STD – Sexual transmitted disease; HTLV - Human T-lymphotropic virus; ChT - Chlamydia trachomatis; BVg – Bacterial vaginosis.*Mestizo refers to people with mixed origin, Indigenous and other, usually living in poor areas of urban centers or along the borders of the rivers deep in the forest.

### 3.1 Rates, access, and screening

Nine manuscripts fell into this category (Table 2). Four studies were conducted in Brazil, and the others were conducted in Honduras, Paraguay, Peru, and Venezuela. One study referred to cervical cancer occurrence: a cancer clinic in São Paulo, Brazil, observed that cervical cancer was the most common neoplasia (48.3 %) in 29 women with solid tumors. The overall 5-year survival rate (men and women) was 40 % (Aguiar et al., 2016). A study from the Brazilian hospital-based cancer registry found, from 2000 to 2012, 65,843 cases of cervical cancer. Of those cases, 143 were in Indigenous women. These women had a 2.38-fold higher likelihood of having late-stage cervical cancer than white women (2.38, 1.06–5.33) (Renna Junior and Silva, 2018).

**Table 2:** Summary of data from the studies included in the category “Rates, Access, and Screening” of the systematic review of the epidemiology of cervical cancer among Indigenous women living in Latin America.

N	Study	Country and Setting	Primary results
7	Pereira (2011) (Pereira et al., 2011)	Brazil, Indigenous Reserve of Dourados, villages of Jaguapirú and Bororó	Overall coverage estimation was 13.9 % (2004), 21.7 % (2005), and 23.5 % in the Jaguapirú community and 8.5 % (2004), 7.2 % (2005), and 5.1 % (2006) in the Bororó community. The estimated coverage of women aged 35–49 years was 28.9 % (2005) and 28.6 % (2006) in the Jaguapirú community, 8.1 % (2005) and 2.2 % (2006) in the Bororó community.
8	Price (2011) (Price and Asgary, 2011)	Honduras, Yamaranguila (western highlands)	88 % of women “have heard” about cervical cytology or Pap tests, and 50 % reported having received one. One-year coverage in the sample was 20 %. Predictors of having had a Pap test included proximity to a municipal center and having a larger number of living children (*p* < 0.01).
16	Fonseca (2015) (Fonseca et al., 2015)	Brazil, Northern Amazonian region (Yanomami Indigenous District; Eastern Indigenous District)	Overall coverage of screening (last three years) was 58 %. Of women older than 25, 83 % reported at least one screening lifetime. Only 3 % of women were aware of their last screening result.
19	Aguiar (2016) (Aguiar et al., 2016)	Brazil, Cancer Clinic, São Paulo	Cervical cancer was the most common neoplasia in women (48.3 %). The five-year cervical cancer survival rate was 40 %. The overall time (for all cancer patients and tumors) from the first symptom to health care was, on average, 9.4 months; the average elapsed time from the first symptom to diagnosis was 9.0 months. The average time from diagnosis to treatment was 3.4 months. Overall, one-third of the patients did not receive treatment (half due to low-performance status and half refused treatment). Overall, 47 % of the patients refused treatment.
21	Renna Jr. (2018) (Renna Junior and Silva, 2018)	Brazil, Hospital-based cancer registries	Indigenous women had a 2.38-fold higher likelihood of having late-stage cervical cancer than white women (2.38, 1.06–5.33).
22	Vargas-Robles (2018) (Vargas-Robles et al., 2018)	Venezuela, Northern part of the Amazon state	77 % of women had never undergone cytological screening.
23	Velazquez (2018) (Velázquez et al., 2018)	Paraguay, Department of Caaguazu, Primary Health Care	51.2 % of women who were screened noted having had a previous screening test.
24	Borges (2019) (Borges et al., 2019)	Brazil, Acre State (Amazonian region)	Cervical cancer (12 C53 cases) and “uterus non-specified cancer” (2 C55 cases) corresponded to the primary causes of cancer death in women (42.4 %), followed by stomach and liver cancers. Overall, cancer mortality in women was similar to what was expected in the North Brazilian Region. The exception was cervical, stomach, and liver cancer, for which an excess of deaths was noted.
25	Collins (2019) (Collins et al., 2019)	Peru, Lower Napo River region of the Peruvian Amazon	Two-thirds of women stated that money was a barrier to accessing healthcare. Other barriers included transportation (54.7 %), childcare (42.7 %), and not wanting to go alone (39.3 %). Roughly half of women (49.4 %) reported they felt they should seek permission to access healthcare. 32.7 % of women had heard of cervical cancer. 70.8 % reported that fear was a barrier to receiving cervical cancer screening, and 53.6 % lacked access to health facilities.

C53 - International Classification of Diseases, C53 Malignant Neoplasm of Cervix Uteri; C55 - International Classification of Diseases, C55 Malignant Neoplasm of Uterus, part unspecified.

A study from Acre State in the northern part of Brazil analyzed cancer deaths in an Indigenous population of mixed-ethnicity individuals. That investigation used the Mortality registry and compared total deaths observed over the same period in the North region (Borges et al., 2019). Cervical cancer was the leading cause of cancer death in women (42.4 %). When comparing the Indigenous and regional population, this study noted excess cervical cancer deaths among Indigenous women (Borges et al., 2019).

This study also noted an average time from first symptoms to treatment of 9.4 ± 9.6 months; the average time elapsed from first symptom to diagnosis was 9.0 ± 8.8 months. The average time from diagnosis to treatment was 3.4 ± 4.6 months. Interestingly, 15 out of 50 patients (30 %) did not receive treatment: eight were not eligible for treatment and received support care, and seven (14 %) refused treatment (Aguiar et al., 2016).

A study from Peru interviewed 121 Indigenous or mixed Indigenous (mestizo) women from the Peruvian Amazon and observed that 75.2 % (88/117) stated that money was a barrier to accessing healthcare. The women also noted transport (54.7 %, 64/117), childcare (42.7 %, 50/117), and not wanting to go alone (39.3 %, 46/117) as being barriers to screening. Additionally, roughly half of the women (53.6 %; 60/112) reported a lack of access to facilities (Collins et al., 2019). In Honduras, in the Yamaranguila municipality, predictors of having had received a Pap smear in a cohort of 134 women included proximity to the municipal center and having many living children (*p* < 0.01) (Price and Asgary, 2011).

Screening coverage was reported in many ways. In a Peruvian study, 50 % of women reported receiving one Pap smear in their lifetime (Price and Asgary, 2011). Among Indigenous women receiving primary health care in the Department of Caaguazu, Paraguay, 51.2 % had received a previous Pap smear (Velázquez et al., 2018). In a cohort of Indigenous women from Peru, 77 % had never received cytological screening (Vargas-Robles et al., 2018). In a group of native women from the Northern Amazon of Brazil over the age of 25, 83 % reported at least one screening event in their lifetime. However, only 3 % were aware of their previous screening result (Fonseca et al., 2015). In Brazil's Indigenous reserve of Dourados, 2.2–28.9 % of women aged 35–49 years had received at least one Pap smear (Pereira et al., 2011).

### 3.2 Prevalence of precursor lesions

Eight of the 15 manuscripts that fell into this category were conducted in Brazil; the others were conducted in Argentina, Peru, Venezuela (two manuscripts), Bolivia, and Paraguay (Table 3). Two Brazilian manuscripts from the same research group studied two Indigenous Districts in the Northern Amazon: the Yanomami and the Eastern District (Fonseca et al., 2015; da Fonseca et al., 2014). The Yanomami are isolated deep in the Amazon, and the Macuxi and Wapishana people have had more consistent contact with non-Indigenous people over the years. The first study in a referral clinic noted a prevalence of cytological high-grade squamous intraepithelial neoplasia (HSIL) of 10.9 % in the Yanomami and 2.5 % in the Eastern District (da Fonseca et al., 2014). The second study accessed women in their communities. The prevalence of cytological HSIL was lower: 0.7 % in the Yanomami and 0.0 % in the Eastern District (Fonseca et al., 2015).

**Table 3:** Summary of data from the studies included in the category “Prevalence of Precursor Lesions” of the systematic review of the epidemiology of cervical cancer among Indigenous women living in Latin America.

	Study	Country and Setting	Primary results
2	Tonon (2004) (Tonon et al., 2004)	Argentina, Region of Missiones, nine Indian settlements	LSIL 3.4 %; HSIL 1.0 %.
3	Brito (2006) (Brito et al., 2006)	Brazil, Pará (Brazilian Amazon), Parakanã community	ASC-US 8.2 %; AGC 6.1 %; LSIL 12.3 %; HSIL 8.2 %.
5	Speck (2009) (de Speck et al., 2015)	Brazil, South Amazon region, Xingu Indigenous Park	2005 and 2007: LSIL 4.1 % and 2.1 %; HSIL 1.5 % and 0.4 %; AS-CUS/AGC 3.9 % and 3.0 %; ASC-H 2.7 % and 0.5 %; Carcinoma 0.4 % and 0 %.
6	Deluca (2011) (Deluca et al., 2011)	Argentina, Formosa	LSIL 7 %; HSIL 0.4 %; CC 0.4 %.
7	Pereira (2011) (Pereira et al., 2011)	Brazil, Indigenous Reserve of Dourados, villages of Jaguapirú and Bororó	Jaguapirú: HPV + LSIL 2.7 %; HSIL 0.6 %. Bororó: HPV + LSIL 1.8 %; HSIL 1.1 %.
9	Blas (2012) (Blas et al., 2012)	Peru, Peruvian Amazon	Any abnormality 11.8 %; LSIL+ 6.9 %.
11	Martorell (2012) (Martorell et al., 2012)	Peru, Loretto Department (Bora native settlement) and Iquitos (urban)	No abnormal results in Bora native women.
13	Fonseca (2014) (da Fonseca et al., 2014)	Brazil, Northern Amazonian region (Indigenous reference center in Boa Vista, Roraima)	Yanomami and Eastern Districts: ASC-US 1.6 % and 1.2 %; LSIL 6.5 % and 1.8 %; HSIL 10.9 % and 2.5 %; Invasive cancer 2.0 % and 0.8 %.
14	Pereira (2014) (Pereira et al., n.d.)	Brazil, São Paulo city, referral Indigenous Clinic of São Paulo Hospital	LSIL 3.3 %; ASC-US 2.2 %; ASC-H 1.1 %; HSIL 1.1 %.
15	Rodrigues (2014) (Rodrigues et al., 2014)	Brazil, Pará e Mato Grosso, Nãncepotiti	ASC-US 3.5 %; 1.2 % ASC—H; 1.2 % AGC; 2.3 % LSIL; 2.3 % HSIL
16	Fonseca (2015) (Fonseca et al., 2015)	Brazil, Northern Amazonian region (Yanomami Indigenous District; Eastern Indigenous District)	Yanomami and Eastern Districts: ASC-US 2.5 % and 1.8 %; LSIL 1.5 % and 0.9 %; HSIL 0.7 % and zero; Invasive cancer 0.4 % and 0 %.
18	Speck (2015) (de Speck et al., 2015)	Brazil, Mato Grosso, Xingu Indigenous Park	Cyto 12–24: ASC-US 3.5 %; ASC-H 1.0 %; LSIL 4.5 %; HSIL 0.5 %; AGC 0.1 %. Cyto >64: ASC-US 0.8 %; ASC-H 2.6 %; LSIL 7.0 %; HSIL 2.6 %; AGC 0 Hysto 12–24: CIN1 0.9 %; CIN2/3 0.4 %; VAIN2 0.1 %. Hysto >64: CIN1 2.6 %; CIN2/3 1.7 %; VAIN2 0.
20	Fuenmayor (2018) (Fuenmayor et al., 2018)	Venezuela, Maniapure region, outpatient clinic	Eñepa women: AS-CUS 2.9 %, LSIL 8.6 %, HSIL 2.9 %.
22	Vargas-Robles (2018) (Vargas-Robles et al., 2018)	Venezuela, Northern part of the Amazon state	(any abnormal result) 9.1 %, 18.2 %, and 4.3 % in women from low, medium, and high community urban indices, respectively (*p* = 0.287).
23	Velazquez (2018) (Velázquez et al., 2018)	Paraguay, Department of Caaguazu, Primary Health Care	ASC-US 10.1 %; LSIL 2.3 %; HSIL 0.8 %

LSIL – Low-Grade Squamous Intraepithelial Neoplasia; HSIL – High-Grade Squamous Intraepithelial Neoplasia; ASC-US – Atypical Squamous Cells of Undetermined Significance; AGC – Atypical Glandular Cells; ASC-H – atypical squamous cells; cannot exclude high-grade squamous intraepithelial lesions; CIN 1, 2, 3 - Cervical Intraepithelial Neoplasia Grades 1, 2, 3; LSIL+ − LSIL or more severe; CC – Cervical Cancer; Cyto – Cytology; VAIN2 – Vaginal Intraepithelial Neoplasia Grade 2.

Two other Brazilian studies focused on the Xingu Indigenous Park (de Gois Speck et al., 2009; de Speck et al., 2015). One manuscript focused on 518 non-previously screened women and found an HSIL prevalence of 1.5 %. Women with abnormal results were referred and treated when necessary. The researchers returned to the same place two years later and found a decrease in HSIL prevalence (i.e., 0.4 %) (de Gois Speck et al., 2009). The other manuscript analyzed cytological results from women in two age groups: 12–24-year-olds and women older than 64. In the youngest group, the prevalence of low-grade squamous intraepithelial neoplasia (LSIL) was 4.5 %; the prevalence of HSIL was 0.5 %. Additional assessments revealed a prevalence of cervical intraepithelial neoplasia Grade 2 or 3 (CIN 2/3) of 0.4 %. Interestingly, the prevalence of LSIL in the older group was 7.0 %, and the prevalence of HSIL was 2.6 %. CIN2/3 was observed in 1.7 % of cases after further assessment (de Speck et al., 2015).

Another Brazilian study conducted in the Indigenous Reserve of Dourados observed an HSIL prevalence of 0.6 % in women of Jaguarinú ethnicity and 1.1 % in women of Bororó ethnicity (Pereira et al., 2011). An Indigenous referral clinic in São Paulo noted a prevalence of HSIL of 1.1 % (Pereira et al., n.d.). In Pará, Panará women, the prevalence of HSIL was 2.3 % (Rodrigues et al., 2014). In the same state, in Parakanã women, the prevalence of LSIL and HSIL was 12.3 % and 8.2 %, respectively (Brito et al., 2006).

In Argentina, a study screened Guarani women across nine Indian settlements and found an HSIL prevalence of 1.0 % (Tonon et al., 2004). In Northern Argentina, a community-based study in Formosa reported a prevalence of 7.0 %, 0.4 %, and 0.4 % for LSIL, HSIL, and cervical cancer, respectively, in Pilagá aboriginal women (Deluca et al., 2012). In Peru, no abnormalities were observed in cytology screenings of 44 Bora native women (Martorell et al., 2012). In 205 cervical cytology samples from Human T-lymphotropic virus (HTLV) negative Shipibo-Konibo women in the Peruvian Amazon, abnormalities were noted in 11.8 % of samples. Furthermore, 6.9 % of the samples harbored LSIL or more severe changes (Blas et al., 2012).

In Venezuela, a study conducted with 35 Indigenous Eñepa ethnicity women in an outpatient clinic revealed an HSIL prevalence of 2.9 % (Fuenmayor et al., 2018). Another study evaluated 89 Piaroa Amerindian women and examined associations between the grade of urbanization and cytological results. The authors found abnormal cytological results in 9.1 %, 18.2 %, and 4.3 % in women from low, medium, and high community urban indices, respectively (*p* = 0.287) (Vargas-Robles et al., 2018). In Paraguay, the prevalence of HSIL was 0.8 % in 129 Indigenous women native to Caaguazu screened at a primary health care center (Velázquez et al., 2018).

### 3.3 HPV prevalence and genotypes

Twelve studies evaluating HPV prevalence, genotypes, and variants were reported in 14 manuscripts. Three of those studies were conducted in Argentina (five manuscripts), three in Brazil, two in Peru, two in Venezuela, and one in Paraguay (two manuscripts) (Table 4). The investigations reported prevalence as “overall HPV prevalence,” referring to low- and high-risk genotypes, and most of them also reported a “high-risk HPV prevalence,” referring to oncogenic genotypes. We followed the authors' definitions of high-risk genotypes.

**Table 4:** Summary of data from the studies included in the category “HPV prevalence and genotypes” of the systematic review of the epidemiology of cervical cancer among Indigenous women living in Latin America.

	Study	Country and Setting	Primary results
**1**	Picconi (2003) (Picconi et al., 2003)	Argentina, Puna and Quebrada region (Jujuy Province)	Most L1 variants belonged to the European branch. 57 % of the E6 samples were variants. LCR variants were 89 % and 69 % European. A new variant was described (AQ-51).
**2, 4**	Tonon (2004) (Tonon et al., 2004) and Tonon (2007) (Tonon et al., 2007)	Argentina, Region of Missiones, nine Indian settlements	**2004:** The overall HPV prevalence was 64 %; 26 % HPV 16/18. hrHPV genotypes included −16, −18, −31, −35, −39, −45, −58, −59, and − 68. 40 % of the HPV cases detected were from the A9 phylogenetic group. **2007:** Of the variants, 51 % were European prototypes. No statistically significant association was found between variants and the grade of cervical lesion.
**3**	Brito (2006) (Brito et al., 2006)	Brazil, Pará (Brazilian Amazon), Parakanã community	hrHPV prevalence of 39.6 %.
**6, 10**	Deluca (2011) (Deluca et al., 2011) and Deluca (2012) (Deluca et al., 2012)	Argentina, Formosa	The overall HPV prevalence was 46.7 %. HPV-16 (19.4 %), −6 and − 18 (5.3 %), −58 (3.5 %), −31 and − 33 (3.1 %). Multiple 12.3 %.
**9**	Blas (2012) (Blas et al., 2012)	Peru, Peruvian Amazon	The prevalence in HTLV0-negative women: any type 29.3 %, hrHPV 22.4 %.
**11**	Martorell (2012) (Martorell et al., 2012)	Peru, Loretto Department (Bora native settlement) and Iquitos (urban)	The overall HPV prevalence in Bora women was 35.4 % (others 27.8–66.8 %), hrHPV 18.9 %. There were no variations in prevalence as a function of age.
**12, 17**	Mendoza (2013) (Mendoza et al., 2013) and Mongelos (2015) (Mongelos et al., 2015)	Paraguay, Department of Presidente Hayes, Indigenous community	The overall HPV prevalence 23.2 %; hrHPV 16.1 % (18.8 % < 30 years, 11.1 % 30–39 years, 8.0 % 40–49 years, 23.1 % > 49 years). hrHPV variantsobserved: −16, −18, −31, −35, −39, −45, −51, −52, −56, −58, −59, −68, −73, and − 82. Single vs. multiple infection hrHPV: 45 % vs. 55 %, HPV -16, −31, and − 45 present in 25 % of multiple infections. 16 (4.4 %), −18 (0.6 %), −31 (2.2 %), −45 (3.3 %), −52 (1.7 %), and − 58 (3.3 %).
**15**	Rodrigues (2014) (Rodrigues et al., 2014)	Brazil, Pará e Mato Grosso, Nãncepotiti	hrHPV prevalence 28.7 %: 11.9 % -16/18/45, 16.7 % other hrHPV.
**16**	Fonseca (2015) (Fonseca et al., 2015)	Brazil, Northern Amazonian region (Yanomami Indigenous District; Eastern Indigenous District)	Y = Yanomami; ED = Eastern District Overall HPV Y 45.9 %; ED 34.5 %. hrHPV Y 34.1 %; ED 19.2 %. HPV -16 Y 9.5 %; ED 2.8 %. HPV -18 = Y 7.2 %; ED 1.9 %. Number of HPV types: Y 42; ED 52. Alpha 9 and 7 more common. hrHPV age-specific curves showing a second peak in Y women after ange of 35–44 years.
**20**	Fuenmayor (2018) (Fuenmayor et al., 2018)	Venezuela, Maniapure region, outpatient clinic	The overall HPV prevalence was 35 %, hrHPV 25 % (28.6 % Eñepa, 20 % Creole). HPV-positive average age in Eñepa 36 years; in Creole, 23 years
**22**	Vargas-Robles (2018) (Vargas-Robles et al., 2018)	Venezuela, Northern part of the Amazon state	The prevalence of hrHPV was 54.5 %, 68.2 %, and 78.3 % in women from low, medium, and high community urban indices, respectively (*p* = 0.237). Alpha diversity was higher in mestizos than in Amerindians from a low urban index.

Legend: HPV - Human papillomavirus; L1 - Major capsid protein of HPV; E6 - Oncoproteins of high-risk HPV; LCR - long control region of HPV; hrHPV – high-risk HPV

The overall prevalence of HPV in the Pilagá aboriginal community in Argentina was 46.7 % (Deluca et al., 2012; Deluca et al., 2011),; it was 64.0 % in Guarani women (Tonon et al., 2004; Tonon et al., 2007). In a study conducted in the Brazilian Amazon, the overall prevalence of HPV was 45.9 % in Yanomami women and 34.5 % in Macuxi and Wapishana women. The Yanomami have had more recent contact with non-Indigenous populations than other Indigenous groups (Fonseca et al., 2015). In Paraguay, the overall prevalence of HPV was 23.2 % in the Department of Presidente Hayes Indigenous community (Mendoza et al., 2013; Mongelos et al., 2015). In Bora women from Peru, the prevalence was 35.4 % (Martorell et al., 2012). In Venezuela, the corresponding prevalence was 35.0 % (Fuenmayor et al., 2018).

The prevalence of hrHPV was 16.1 % in Hayes, Argentina (Mendoza et al., 2013; Mongelos et al., 2015). In Brazil, it was found to be 34.1 % in Yanomami women and 19.2 % in Macuxi and Wapishana women (Fonseca et al., 2015). Another study found an hrHPV prevalence of 28.7 % in Panará women from Brazil Central (Rodrigues et al., 2014). In the Brazilian Amazon state of Pará, the prevalence of hrHPV was 39.6 % (Brito et al., 2006). In Peru, in Bora women, the prevalence of hrHPV was 18.9 %; in Shipibo-Konibo HTLV-negative women, the corresponding prevalence was 22.4 % (Martorell et al., 2012; Blas et al., 2012). In Venezuela, the prevalence of hrHPV was 25.0 % in Eñepa and Creole women and 54.5 %, 68.2 %, and 78.3 % in Piaroa Amerindian women from low, medium, or high urbanization grades, respectively (Vargas-Robles et al., 2018; Fuenmayor et al., 2018). In Paraguay, the prevalence of hrHPV was 16.1 % (Mendoza et al., 2013; Mongelos et al., 2015).

In a study of women in the Argentinian Pilagá community, the most frequent hrHPV genotypes were hrHPV-16 (19.4 %), hrHPV-18 (5.3 %), hrHPV-58 (3.5 %), hrHPV-31 (3.1 %), and hrHPV-33 (3.1 %). Multiple infections were found in 12.3 % of the cohort (Deluca et al., 2012; Deluca et al., 2011). In Guarani Argentinean women, the hrHPV genotypes observed included hrHPV-16, hrHPV-18, hrHPV-31, hrHPV-35, hrHPV-39, hrHPV-45, hrHPV-58, hrHPV-59, and hrHPV-68. In this study, 40 % of overall HPV cases were from the A9 phylogenetic group (viral types 16, 58, 31, and 35) (Tonon et al., 2004; Tonon et al., 2007).

In the Brazilian Amazon study, the prevalence of HPV-16 in Yanomami and Macuxi/Wapishana women was 9.5 % and 2.8 %, respectively; the prevalence of HPV-18 was 7.2 % and 1.9 %, respectively (Fonseca et al., 2015). In this study, over 50 types of HPV were noted; the more common phylogenetic groups were alpha 9 and 7. hrHPV age-specific curves revealed a second peak in Yanomami women after an age of 35–44 years (Fonseca et al., 2015). In Brazil central, the prevalence of HPV-16/18/45 was 11.9 %; the prevalence of hrHPV was 16.7 % (Rodrigues et al., 2014).

In Paraguay, the age-specific prevalence of hrHPV was 18.8 % in women younger than 30 years, 11.1 % in women ranging in age from 30 to 39 years, 8.0 % in women ranging in age from 40 to 49 years, and 23.1 % in women over an age of 49 years. Single or multiple hrHPV infections were noted in 45.0 and 55.0 % of cases, respectively; HPV-16, HPV-31, and HPV-45 were present in 25 % of various infections. The prevalence of HPV-16, HPV-18, HPV-31, HPV-45, HPV-52, and HPV-58 was 4.4 %, 0.6 %, 2.2 %, 3.3 %, 1.7 %, and 3.3 %, respectively (Mendoza et al., 2013; Mongelos et al., 2015).

Two Argentinean studies focused on HPV-16 variants. Among HPV-16 positive samples from Jujuy Province in Argentina, one study found that most L1 variants belonged to the European branch (Picconi et al., 2003). Most non-European variants, accounting for 10 % of the sample, were Asian-American. Roughly half (57 %) of the E6 samples were variants, and they were detected mainly in squamous intraepithelial lesions and invasive cervical cancer (Picconi et al., 2003). The long control region (LCR) variants constituted 89 % of the samples overall and 69 % of the European samples. A new variant was described in this study: AQ-51, Argentine Quechua-51, probably from the Asian-American branch (Picconi et al., 2003). Of the variants in the Region of Missiones, 51 % were from the European prototype. No statistically significant association was found between variants and cervical lesion grades (Tonon et al., 2007).

In Peru, no changes in hrHPV prevalence were observed as a function of patient age (Martorell et al., 2012). In Venezuela, the average age of HPV-positive Eñepa women was 36 years; the average age of HPV-positive women belonging to the Creole group was 23 years (Fuenmayor et al., 2018). In Piaroa Venezuelan Amerindians, differences in the frequency of single or multiple HPV infections were not significant between urban groups; the alpha diversity (variety of microbes) was higher in mestizos than in Amerindians from a low urban index (Vargas-Robles et al., 2018).

### 3.4 HPV coinfections

Three studies provided consistent information about HPV coinfections. One study was conducted in Paraguay (two manuscripts), one was conducted in Argentina, and one was conducted in Peru (Table 5). The study from Paraguay included 181 women of different ethnicities from the Department of Presidente Hayes Indigenous community (Mendoza et al., 2013; Mongelos et al., 2015). The overall prevalence and prevalence of coinfection with HPV were: syphilis at 11.6 % and 7.1 %; *Trichomonas vaginalis* at 10.5 % and 11.9 %; *Chlamydia trachomatis* (ChT) at 9.9 % and 21.4 %; HIV at 0.6 % and none; *Gardnerella vaginalis* at 45.9 % and 47.6 %; *Mycoplasma hominis* at 30.9 % and 38.1 %; *Ureaplasma urealyticum* at 20.4 % and 26.2 %; and *Cândida* sp. At 7.2 % and 4.8 %.

**Table 5:** Summary of data from the studies included in the category “HPV coinfections” of the systematic review of the epidemiology of cervical cancer among Indigenous women living in Latin America.

	Study	Country and Setting	Primary results
6	Deluca (2011) (Mendoza et al., 2013)	Argentina, Formosa	The prevalence of ChT is higher in HPV-positive individuals (34.9 %, 2.28, 1.20–4.38, *p* = 0.007). The prevalence in hrHPV was 33.8 %, low-risk HPV 29.1 %; single HPV infection 33.3 % and multiple HPV infections 39.3 %.
10	Blas (2012) (Deluca et al., 2012)	Peru, Peruvian Amazon	HTLV-1 infection was associated with an HPV infection of any type (PR 2.10, 1.53–2.87) and hrHPV infection (PR 1.93, 1.04–3.59). HTLV-2 infection was not significantly associated with HPV infection. HTLV-1 or 2 were not associated with any abnormal cytology.
12, 17	Mendoza (2013) (Mendoza et al., 2013) and Mongelos (2015) (Mongelos et al., 2015)	Paraguay, Department of Presidente Hayes, Indigenous community	The overall and co-infection with HPV prevalence was: syphilis 11.6 % and 7.1 %, TVg 10.5 % and 11.9 %, ChT 9.9 % and 21.4 %, HIV 0.6 % and none, GVg 45.9 % and 47.6 %, *Mycoplasma hominis* 30.9 % and 38.1 %, *Ureaplasma urealyticum* 20.4 % and 26.2 %, and *Cândida* sp. 7.2 % and 4.8 %. The co-infection rate of ChT and HPV-positive samples significantly differed from that of HPV-negative samples (21.4 % positive vs negative 6.5 %, *p* = 0.004). A normal Nugent Score (0–3) was observed in 27.5 % of hrHPV-positive and 55.3 % of hrHPV-negative cases (*p* = 0.006); a Nugent Score suggestive of BVg was noted in 51.7 % of hrHPV-positive cases and 27.6 % of hrHPV-negative cases (*p* = 0.01)

Legend: ChT - Chlamydia trachomatis; HPV - Human papillomavirus; hrHPV – high-risk HPV; HTLV - Human T-lymphotropic virus; PR – Prevalence ratio; TVg - Trichomonas vaginalis; HIV - Human immunodeficiency virus; GVg – Gardnerella vaginalis

The coinfection rate of ChT and HPV significantly differed from that of the non-co-infection rate (21.4 % positive versus negative 6.5 %; *p* = 0.004). A Nugent score suggestive of Bacterial Vaginosis (7–10) was noted in 51.7 % of hrHPV-positive cases and 27.6 % of hrHPV-negative cases (*p* = 0.01) (Mendoza et al., 2013; Mongelos et al., 2015).

In the Argentinian study of 227 Pilagá aboriginal women in Formosa, the prevalence of ChT was higher in HPV-positive women than in HPV-negative women (34.9 % and 19.0 %, respectively; OR 2.28, CI 1.20–4.38, *p* = 0.007) (Deluca et al., 2011). Those authors found that HTLV-1 infections were associated with hrHPV infections (PR 1.93, 1.04–3.59). HTLV-2 infections were not significantly associated with HPV (Blas et al., 2012).

## 4 Discussion

High rates of cervical cancer have been observed in Indigenous women living in Latin America (Moore et al., 2014; Borges et al., 2019; Aguiar et al., 2016; Forman et al., 2012). The studies examined in this review support this fact (Borges et al., 2019; Aguiar et al., 2016; Renna Junior and Silva, 2018). Diagnoses at late stages and low survival rates (Aguiar et al., 2016; Renna Junior and Silva, 2018) among Indigenous women highlight disparities in cancer outcomes and the need for targeted interventions to improve screening and care.

The prevalence of HSIL was not consistently high in the studies we examined. That finding is similar to what has been observed in the general population of Latin America (Vale et al., 2014; Levi et al., 2019; Campaner and Fernandes, 2023). Geographic isolation and screening activities may influence the prevalence of HSIL. In a referral clinic, the cytological prevalence of HSIL was high in Yanomami Brazilian women, an ethnicity with recent contact with the non-Indigenous population (da Fonseca et al., 2014). In Xingu Indigenous Park, non-previously screened women exhibited a reduction in HSIL prevalence after two years of screening activities (de Gois Speck et al., 2009). Among Venezuelan Piaroa women, the prevalence of HSIL increased with a decreasing urbanization index (Vargas-Robles et al., 2018).

The discrepancy between the non-elevated rates of precursor lesions and the high rates of cervical cancer among Indigenous women may be explained by a lack of access to diagnosis and treatment after a suspected precursor lesion. Indigenous women are more likely to experience delays in their diagnosis and treatment (Aguiar et al., 2016). Cultural behaviors may contribute partly to barriers to access to health care, as observed in this review (Collins et al., 2019; Price and Asgary, 2011) and pointed out by the literature (Kolahdooz et al., 2014; Han et al., 2024). However, the fragility of local health systems in regions where Indigenous women live seems to be the cornerstone issue. Public policies on cervical cancer should consider Indigenous women a vulnerable population (Moore et al., 2014; Muslin, 2024; Han et al., 2024; de Melo et al., 2024).

We found that the overall prevalence of HPV was high, over 30 %, in most studies (Fonseca et al., 2015; Vargas-Robles et al., 2018; Tonon et al., 2004; Martorell et al., 2012; Blas et al., 2012; Fuenmayor et al., 2018; Deluca et al., 2011). The overall global prevalence of HPV has been estimated to be 11.7 % and 16.1 % in Latin America (Bruni et al., 2010; Walmer et al., 2013). The prevalence of hrHPV types varied from 16.1 to 28.7 % of women (Fonseca et al., 2015; Vargas-Robles et al., 2018; Rodrigues et al., 2014; Brito et al., 2006; Tonon et al., 2004; Martorell et al., 2012; Blas et al., 2012; Fuenmayor et al., 2018; Deluca et al., 2011; Mendoza et al., 2013). Some studies have reported populations with very high hrHPV rates, such as Yanomami (34.1 %) (Fonseca et al., 2015) and Parakanã women (39.6 %) (Brito et al., 2006) in Brazil and Piaroa Amerindian women in Venezuela (over 54.4 %) (Vargas-Robles et al., 2018; Fuenmayor et al., 2018).

The most frequent hrHPV genotypes did not differ from those of the general population (Bruni et al., 2010). The prevalence of HPV-16 varied from 4.4 to 19.4 % (Fonseca et al., 2015; Rodrigues et al., 2014; Deluca et al., 2011). In the general population, about 22.5 % of HPV infections are HPV-16 (Bruni et al., 2010). A second peak of hrHPV was observed in Yanomami women above an age of 35–44 years (Fonseca et al., 2015) and in women over 49 years old in an Indigenous community in Paraguay (Mendoza et al., 2013; Mongelos et al., 2015). This second peak is well-described worldwide in regions where screening is deficient (Forman et al., 2012; Bruni et al., 2010). This pattern of two peaks of prevalence is multifactorial and may be due to immunosenescence, perimenopausal immunity changes, or reactivation of a latent infection (Forman et al., 2012; Bruni et al., 2010).

We found that ChT was significantly associated with HPV in two studies (Deluca et al., 2011; Mendoza et al., 2013; Mongelos et al., 2015). This finding is consistent with the literature noting that other sexually transmitted diseases are associated with HPV in the general population (Mancini et al., 2018; Panatto et al., 2015; Suehiro et al., 2021).

Several of the studies noted the early age at which both Indigenous and non-Indigenous women are screened for HPV. It is important to state that screening is opportunistic in Latin America. Up until the last decade, recommendations of when to start screening were more linked to the initiation of sexual activity than to age. When data were stratified by age groups in the studies, we prioritized reporting data in women older than 25.

There are several limitations to this review. For starters, most of the study designs were designed by non-Indigenous people. The analysis might be accordingly biased. Unfortunately, no Indigenous people were part of the team that conducted this review. However, two authors have clinical experience either providing care to Indigenous women (IR and DBV) or supervising Indigenous students (FS and DBV). Additionally, we did not include qualitative data. Furthermore, precursor lesions were generally estimated based on cytological results rather than histology of cervical biopsies. The prevalence of HPV was estimated based on multiple tests, and the length of the time period analyzed could have impacted our results because test technology has improved significantly in recent years. We also did not include vaccine data because most Latin American countries had not yet implemented this practice during the study period. Health systems are expected to face the same challenges in implementing vaccination as improving access to screening, diagnosis, and treatment. Despite these limitations, this systematic review still provides useful information.

## 5 Conclusions

This review showed that cervical cancer is the most common type of cancer to affect Latin American Indigenous women; diagnoses are typically made at late cancer stages. The prevalence of precursor lesions was higher in women who were more geographically isolated and in women who had had more recent contact with non-Indigenous people. Screening appears to improve outcomes. However, Indigenous women may experience delays in their diagnosis and treatment. Some studies reported populations with a very high prevalence of hrHPV, and the most frequent genotypes noted did not differ from those of the general population.

Strategies aimed at eliminating cervical cancer should focus on improving access to screening and prompt treatment of precursor lesions among Indigenous women. Early diagnosis and treatment of invasive lesions are imperative for improving survival rates and quality of life, both of which are critical to preserving the cultural values of this population.

## Author statement

This is part of a study approved by the University of Campinas Ethics Committee through the Brazilian National Platform (CAAE: 51446421.9.0000.5404). It was registered in PROSPERO (CRD42021287229).

## CRediT authorship contribution statement

**Iria Riberio Novais:** Writing – review & editing, Writing – original draft, Formal analysis, Data curation, Conceptualization. **Camila Olegario Coelho:** Writing – review & editing, Writing – original draft, Methodology, Formal analysis, Data curation. **Carla Fabrine Carvalho:** Writing – review & editing, Writing – original draft. **Fernanda Surita:** Writing – review & editing, Validation. **Diama Bhadra Vale:** Writing – review & editing, Writing – original draft, Validation, Supervision, Project administration, Methodology, Investigation, Formal analysis, Data curation, Conceptualization.

## Declaration of competing interest

The authors declare that they have no known competing financial interests or personal relationships that could have appeared to influence the work reported in this paper.

## Data Availability

Data will be made available on request.
